# Patient Isolation Precautions: Are They Worth It?

**DOI:** 10.1155/2016/5352625

**Published:** 2016-04-12

**Authors:** Elliott Sprague, Steven Reynolds, Peter Brindley

**Affiliations:** ^1^University of Alberta, Edmonton, AB, Canada T6G 2B7; ^2^Critical Care Medicine, Fraser Health, New Westminster, BC, Canada V3L 3W7; ^3^Department of Medicine (Infectious Diseases and Intensive Care), University of British Columbia, Vancouver, BC, Canada V5Z 1M9; ^4^Critical Care Medicine, Anesthesiology and Medical Ethics, University of Alberta, Edmonton, AB, Canada T6G 2B7

## Abstract

Isolation precautions are intended to minimize pathogen transmission and reduce hospital-acquired infections. More recently, the effectiveness of isolation precautions has been questioned because of increasing evidence of risks. These putative downsides are divided into a quantifiable monetary cost (i.e., a literal cost to the system) and clinically important but less easily quantifiable costs (i.e., “costs” to the patient). The authors also briefly review deisolation and alternatives to isolation. The present review is not arguing against appropriate isolation or precautions, simply that the authors consider both risks and benefits and disseminate up-to-date information. Their patient-focused goal is to mitigate risks for those who truly need isolating and to end isolation as soon as it is safe and appropriate to do so.

## 1. Introduction

Isolation precautions (IPs) are used to minimize pathogen transmission and hospital-acquired infections. The three main indications are (i) microorganisms with antibiotic resistance (e.g., Methicillin-Resistant* Staphylococcus aureus* (MRSA), Vancomycin-Resistant* Enterococcus* (VRE), and Extended Spectrum Beta-Lactamase (ESBL) secreting organisms), (ii) microorganisms with high transmission (e.g.,* Clostridium difficile* (C Diff),* Mycobacterium tuberculosis* (TB), norovirus, and influenza virus), and (iii) microorganisms with high virulence (e.g. severe acute respiratory syndrome (SARS) and Ebola virus disease (EVD)).

It may be difficult to compare different microorganisms and different IPs (Table 1). Regardless, guidelines suggest that IPs “work”; namely, their use is associated with reduced transmission and lower morbidity [1–3]. Accordingly, IPs are widespread and widely supported. Current guidelines are generally accepted as intuitive, and older studies have found that contact precautions can prevent MRSA infections and are cost effective [4]. However, in 2004, a British Medical Journal review [5] concluded that the issue is not straightforward. Specifically, while IPs have the potential to reduce transmission, there is conflicting data about benefits versus harms. Regardless, there is room for the debate and the need for more study.

There are many putative downsides to isolation that must be balanced against putative benefits. These include a quantifiable monetary cost (i.e., a literal cost to the system), as well as clinically important but less easily quantifiable costs (i.e., “costs” to the patient), and these are the subject of this review. This paper is not arguing against hand-washing, nor are we claiming that IPs definitively* cause* worse outcomes. However, with microorganisms such as MRSA and VRE, there is a growing association between IPs and increased complications (see below). We are also not arguing against IPs for virulent microorganisms, such as EVD, though authors have questioned whether concerns are overblown [6, 7]. Instead, knowing that IPs also have downsides is clinically relevant to mitigating risks for those patients who truly need isolation and to ending isolation as soon as it is safe to do so. The goal is to optimize patient safety while also promoting patient-centered care.

## 2. The Monetary Cost of Patient Isolation to the System

We expend finite resources whenever we screen and isolate. However, quantifying precise dollar amounts is difficult. This is because of so many variables: microorganisms differ; screening methods differ, and isolation equipment differs (Table 1). There are also potentially “hidden costs” such as labour-time for HCWs (i.e., time donning and doffing protective gear). There is the cost of employing infection control practitioners. There are also the cost of follow-up and the cost of repeat testing, as well as the inability to locate isolated and nonisolated patients in the same room.

There may be unfactored costs such as delayed discharge, preventable ICU-days, and postponed surgeries. While having a resistant organism may be associated with increased patient frailty or disease burden, patients on IPs remained in tertiary care centers longer while awaiting transfer: mean of 10.9 days versus 4.3 days [9]. Where there is literature regarding cost, it has focused on MRSA and VRE. With these two microorganisms, the mean cost associated with isolation ranges from $400–$2000 per positive-patient per day [10]. It has also been estimated that Canadian EVD precautions and preparations have exceeded 90 million dollars and countless hours, without a single case to date [11].

## 3. Other “Costs” of Patient Isolation

Patients may also currently “pay a price” when isolated. For example, regarding whether IPs result in lower quality patient care, the data does not show clear causation but does suggest negative associations. A 2003 JAMA study found that isolated patients were twice as likely to experience an adverse event during hospitalization (31 versus 15 adverse events per 1000 days; *P* < 0.001) and seven times more likely to experience a* preventable* adverse event (20 versus 3 adverse events per 1000 days; *P* < 0.001) [12]. Adverse events included increased falls, pressure ulcers, and fluid and electrolyte errors. Isolation may also be associated with decreased patient satisfaction. For example, there were higher rates of formal complaints towards the institution: 8% of isolated patients and less than 1% of nonisolated patients.

When compared to nonisolated patients, isolated patients receive less attention from healthcare workers (HCWs). This includes, on average, approximately 50% less room entries, 50% less time spent in their rooms, and 50% less physical contact. Nurses failed to record vital signs as frequently, and physicians provided a recorded progress note half as often. IP patients were also half as likely to be examined by attending physicians and received, on average, 25% less time from interns [13]. Clearly, HCWs need to redouble efforts with isolated patients [12].

IP patients also have 23% less contact from visitors compared to nonisolated patients [14]. Chronic illness is already associated with feeling socially isolated [15]. However, well-intentioned IPs may compound social isolation with literal isolation. While data is limited, IPs might increase the patient's sense of vulnerability at a time when most people crave social connection. While speculative, isolation might even make patients feel they are “unclean” or even “undeserving attention.” A provocative 2015 New England Journal of Medicine (NEJM) editorial also suggests the threat of quarantine could deter patients from seeking help [7].

Several studies have shown that isolated patients have increased rates of depression. The largest of these followed up over 70,000 patients for more than two years [16]. Day et al. found that, in the non-Intensive Care Unit (ICU) setting, depression was 40% more prevalent in patients on contact precautions. In contrast, this study found no association between depression and being admitted to ICU. In fact, the increased HCW contact associated with ICU admission (typically never less than one nurse for two patients) and more frequent assessments (typically never less than vital signs every four hours) may mitigate against ICU depression. Regardless, it reemphasizes that our patients need more than just our cognitive abilities: they need to feel cared for.

IPs may also be associated with increased rates of delirium. This could be due to increased illness severity in patients who are isolated. However, a 2012 study [17], which reviewed over 60,000 admissions, found that patients under contact precautions had delirium rates that were not just slightly increased but more than double that of the control: 16.1% versus 7.6%. Moreover, the association between isolation and delirium persisted even after adjusting for potential confounders such as comorbid condition, age, sex, ICU status, and length of hospitalization. Isolated patients also had increased length-of-stay plus higher usage of antipsychotics and physical restraints. Delirium is known to be associated with increased morbidity and mortality [18]. By decreasing isolation as soon as it is appropriate to do so, we may protect patients from avoidable complications.

## 4. So Is Isolation Worth It?

HCWs understand that their job involves weighing costs (the expenditure of finite resources, etc.) against benefits (keeping other patients safe, etc.). Provocative new research challenges what may have previously seemed to be self-evident. MRSA IPs are intended to decrease the spread to noncolonized patients and the frequency of MRSA-related infections. Getting the balance right matters because MRSA is the most frequently isolated pathogen, with up to 10% of tertiary care patients colonized [19]. However, in both ward and ICU settings, data suggests that MRSA screening, isolation, and contact precautions do not convincingly achieve these goals [20]. For example, a 2011 NEJM cluster-randomized ICU study found no significant change in the rate of MRSA colonization and MRSA-related infections with and without expanded barrier precautions: 16.0% versus 13.5%, *P* = 0.39 [19].

The aforementioned article [19] also found that VRE colonization, infection, and spread were not decreased in ICU patients after culture-based active surveillance and expanded barrier precautions. There was also no increase in the control group. The lack of benefit from IPs was surprising because surveillance identified a sizable subgroup of colonized patients who would not otherwise have been recognized. The evidence is mixed in the non-ICU setting, but again, in light of more recent studies, there is no longer an overwhelming signal that IPs achieve their goal.

There is less evidence to support IPs for VRE compared to MRSA, although VRE has received far less study. Accordingly, there is even less literature to support (or refute) IPs for* C difficile* and respiratory viruses. Regardless, it does appear that patients commonly remain on isolation when benefits no longer outweigh risks [19]. This may be because frontline clinicians (understandably) err towards overisolating not underisolating. Alternatively, there may be inadequate knowledge, or guidelines, regarding when to deisolate patients (Table 1). Regardless, inconsistent application of IPs might erode trust in, and compliance with, the healthcare system. For example, IPs may be difficult to fastidiously apply in the Emergency Department and are usually removed upon hospital discharge.

## 5. When Is It Appropriate to Deisolate Patients?

Regarding discontinuation of MRSA and VRE isolation, the evidence is unfortunately limited. However, a single document, based mostly on expert opinion and published in 1995 by the Centre of Disease Control Healthcare Infection Control Practises Advisory Committee, stated that in order to discontinue isolation there should be three negative nasal swabs for MRSA each separated by one week. Similarly, they advised three negative rectal swabs for VRE, also separated by one week [21].

Subsequently, in 2002, Byers et al. performed a retrospective cohort study of VRE colonization (*n* = 116). They concluded that of the 64% who became VRE swab-negative, 92% were still negative on first follow-up swab and 95% were negative on both the second and third follow-up swab [22]. This begs the currently unanswered question of whether the second and third follow-up swabs help or hinder. In other words, are additional negative swabs a useful precaution or an unnecessary delay?

In 2014, regarding both MRSA and VRE colonization, Ghosh et al. [23] found that in 365 patients who were initially positive for either (but not both) microorganism and were also hospitalized over 30 days, 11% became MRSA negative and 18% VRE negative [23]. They estimated that this resulted in saving 2152 patient-days of patient precautions over one year, and therefore that reswabbing is cost-effective.

## 6. Are There Alternatives to Isolation?

The 2013 REDUCE MRSA study [8] argues that a better approach to MRSA is decolonization: using mupirocin and chlorhexidine. Huang et al. compared ICU patients and three approaches: (i)* isolating* those patients colonized with MRSA (*n* = 23,480); (ii)* decolonizing* those patients colonized with MRSA (*n* = 22,105); and (iii) universal decolonization without checking MRSA status (*n* = 26,024). Universal decolonization resulted in decreased transmission and a significant reduction in* all* bloodstream infections (not just MRSA), compared to either targeted decolonization or screening with isolation. This approach also eliminated costly MRSA admission screening (approximately 50 dollars per patient [9]) and all the costs of isolation mentioned above.

Universal decolonization reduced MRSA positive cultures by 37%, reduced bloodstream infections by any pathogen by 44%, and prevented one bloodstream infection per 99 patients. Notably, mupirocin resistance was not studied; however, other side effects were trivial: a mild skin irritation, in only seven patients. In contrast to MRSA, both a 2012 Canadian systematic review and a 2014 Lancet cluster-randomized trial article concluded that there was no reduction in transmission or infection following VRE or ESBL decolonization, compared with no decolonization [24, 25] Overall, studies have not yet led to widespread decolonization.

Authors have concluded that we need better education for HCWs, we need better explanation to patients and family members, and, where possible, we need to avoid having nurses to concomitantly look after isolated and nonisolated patients. According to Butterfield [26], there is also the possibility of carefully “watching the patient in isolation without gowning and gloving, that is to say, marking off an area just inside the patient's room that can be entered without precautions.” Regardless, the issue of isolation and deisolation warrants our continued attention.

## 7. In Closing

This short review is not a call to abandon IPs nor to ignore IPs once in place. However, it seems that the issue of IPs is not clear-cut, that guidelines need regular review, and that we need a mechanism for dissemination if new evidence becomes available. At the same time as redoubling efforts to improve hand hygiene, we could also highlight the potential negative effects of inappropriate IPs. Finally, hospitals have their own policies for implementing and removing precautions. A more unified approach could help frontline workers, could standardize data collection, and might increase efficiency and throughput.

## Figures and Tables

**Table 1:** Typical isolation precautions and deisolation recommendations for various microorganisms. Adapted from [3] Siegel et al. and [8] Huang et al.

Organism	Indication for isolation	Precautions	Indication for isolation	Removal of isolation
*Methicillin-Resistant Staphylococcus Aureus (MRSA)*	Antibiotic resistance	Contact	Positive screening swab (by culture or nucleic acid testing [NAT]) or evidence of active infection	Usually after 3 negative swabs at 1-week intervals and off MRSA antibiotics × 72 hrs prior to testing

*Vancomycin-Resistant Enterococcus (VRE)*	Antibiotic resistance	Contact	Positive screening swab (by culture or nucleic acid testing) or evidence of active infection	Usually after 3 negative swabs at 1-week intervals and off VRE antibiotics × 72 hrs prior to testing

*Extended Spectrum Beta-Lactamase (ESBL)*	Antibiotic resistance	Contact	Culture of ESBL-secreting organisms	Usually for duration of hospitalization

*Clostridium difficile*	Propensity for transmission	Contact	Liquid stool positive for toxin	Usually after symptom resolution × 48 hrs (negative test not usually required)

*Norovirus*	Propensity for transmission	Contact	Diarrhea in patient with suspected outbreak exposure or positive culture	Usually following resolution of symptoms

*Influenza*	Propensity for transmission	Droplet	Influenza-like illness defined as acute respiratory infection; temperature ≥ 38°C; cough within 10 days	Usually following negative testing or after 72 hours of antiviral therapy

*Tuberculosis (TB)*	Propensity for transmission and antibiotic resistance	Airborne	Known TB, epidemiologic risk factor(s) for TB infection with compatible clinical syndrome	Usually requiring clearance by TB services

*Ebola virus*	Emerging pathogen and potential for transmission	Droplet and airborne	Known active infection (positive by NAT or serology) or epidemiologic risk (fever within 21 days of travel from Ebola endemic area)	Usually following negative polymerase chain reaction testing from blood collected within 72 h

Contact Precautions: gown and gloves for staff and visitors.

Droplet Precautions: gown, gloves, surgical mask, and eye protection.

Airborne Precautions: gown, gloves, and fit-tested N-95 mask.
